# A visualization analysis of research on arterial compression hemostatic devices using VOSviewer and CiteSpace

**DOI:** 10.3389/fneur.2025.1540909

**Published:** 2025-03-05

**Authors:** Li Liu, Juan Xiong, Jianmei Xu, Ping Tu

**Affiliations:** ^1^Department of Neurosurgery, The Second Affiliated Hospital, Jiangxi Medical College, Nanchang University, Nanchang, Jiangxi, China; ^2^School of Nursing, Jiangxi Medical College, Nanchang University, Nanchang, Jiangxi, China; ^3^Post Anesthesia Care Unit, The Second Affiliated Hospital, Jiangxi Medical College, Nanchang University, Nanchang, Jiangxi, China

**Keywords:** radial artery, nursing, hemostasis, compression, cluster analysis, visual analysis

## Abstract

**Background:**

Cardiovascular and cerebrovascular diseases pose a significant health challenge in modern society, with the advancement of interventional therapy and vascular intervention technology playing crucial roles. In the context of post-interventional procedures, the application of suitable pressure at the puncture site is of utmost importance for achieving hemostasis. A variety of arterial compression devices are utilized in clinical settings to facilitate this critical step. A bibliometric analysis is used to assess the impact of research in a particular field. This study seeks to explore the research trends, key themes, and future directions of arterial compression hemostatic devices in international scholarly literature to inform future research endeavors.

**Methods:**

English-language literature on arterial compression hemostatic devices was systematically retrieved from the Web of Science (WOS) and Scopus databases until December 31, 2024. In this study, we employed VOSviewer 1.6.18 and CiteSpace 6.2.r4 to systematically analyze a comprehensive set of parameters, which included authorship and institutional affiliations, geographical distribution by country, and thematic categorization through keywords.

**Results:**

In total, 4,358 relevant publications were retrieved. This study’s results section highlights a growing body of research on arterial compression hemostasis devices, with a significant increase in publications post-2000, reaching 107 in 2022. Department of Cardiology leads in institutional contributions, while ‘Bernat, lvo’ is the most prolific authors. Keyword analysis identifies “human,” “article,” “hemostasis,” “female,” and “male” as key terms, with 7 thematic clusters revealed by hierarchical clustering.

**Conclusion:**

The results provide an overview of research on arterial compression hemostatic devices, which may help researchers better understand classical research, historical developments, and new discoveries, as well as providing ideas for future research.

## 1 Introduction

In recent years, China’s economic and cultural development has significantly improved living standards (1, 2)_._ However, cardiovascular and cerebrovascular diseases remain a major health concern (3–5). Advances in medical technology have led to the widespread use of interventional treatments (6–8), which have shown positive clinical outcomes. These diseases are the primary focus of interventional procedures, with vascular intervention techniques becoming increasingly important (9). Inadequate post-treatment compression hemostasis often leads to complications, highlighting the need for effective arterial compression hemostasis devices. This has driven their clinical application and increased research interest in this area. To improve arterial hemostasis and reduce complications, various devices are used in clinical practice. Examples include modified chitosan hemostatic sponges for radial artery hemostasis after coronary interventional therapy (10, 11), and individualized controlled pressure infusion methods for post-radial artery coronary interventional procedures (12). This study uses bibliometric and visual analysis to evaluate existing English literature on arterial compression hemostasis devices (13–16). The aim is to understand current research trends, publication patterns, and future research directions. It also explores collaborative networks among institutions and researchers to provide insights for future studies (17).

## 2 Data sources and research methods

### 2.1 Data sources

**Figure 1:**
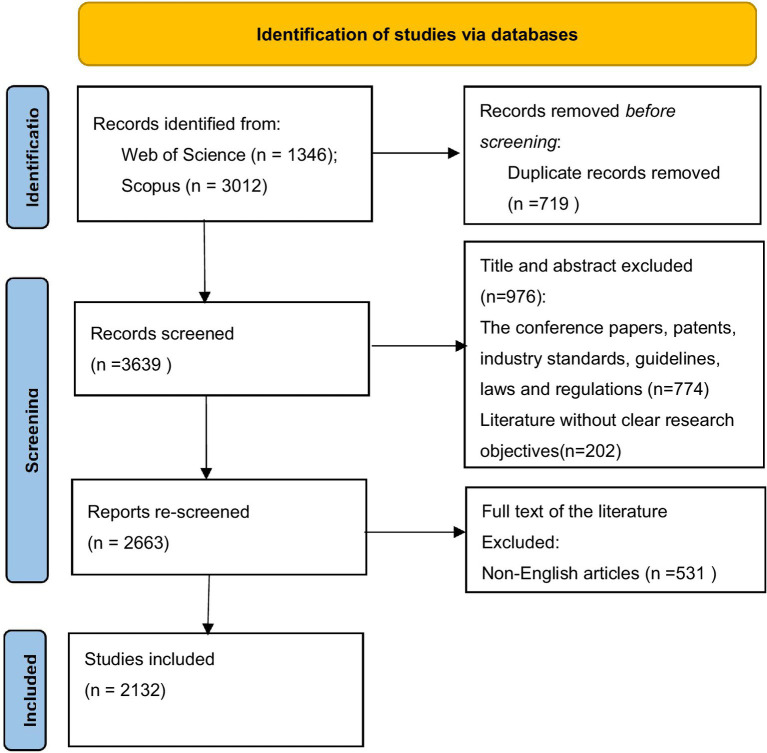
Flow chart of the literature review process.

Data acquisition for this study was conducted utilizing the prestigious Web of Science (WOS) and Scopus databases to ensure the credibility and scope of the data. A search strategy including terms such as Arter*, “Hemosta*,” “stopping bleeding,” “closure,” and “Compressor” or “device” was employed, resulting in a total of 4,358 records retrieved. After careful adjustments and refinement of the search strategy, 2,132 records were deemed relevant and included in the study. The search period spanned from the inception of the database to 2024-12-31, with a focus on online published literature pertaining to arterial compression hemostatic devices or equipment. The specific literature screening process is detailed in Figure 1.

Inclusion criteria for literature encompassed online publications specifically related to the aforementioned topics. Literature types are limited to “Article” and “Review,” while exclusion criteria included duplicate publications, conference papers, patents, industry standards, guidelines, laws, regulations, and literature without clear research objectives. An extensive screening process was carried out by two independent researchers who reviewed titles, abstracts, and extracted relevant data, resolving any discrepancies through collaborative discussions.

### 2.2 Statistical analysis

The selected literature was organized and managed using EndNote X8 software, with relevant data imported into VOSviewer 1.6.18 and CiteSpace 6.2.r4 for the visualization of co-occurrences and burst detection of institutions, authors, and keywords within the research domain of arterial compression hemostatic devices. Parameters were meticulously adjusted to generate a knowledge map for cluster analysis, enabling a comprehensive overview of research trends and associations (18, 19). Bibliometric analysis methods were leveraged for the statistical and analytical management of data, facilitating a thorough examination and interpretation of the collected information (20).

## 3 Results

### 3.1 Analysis of publication volume

**Figure 2:**
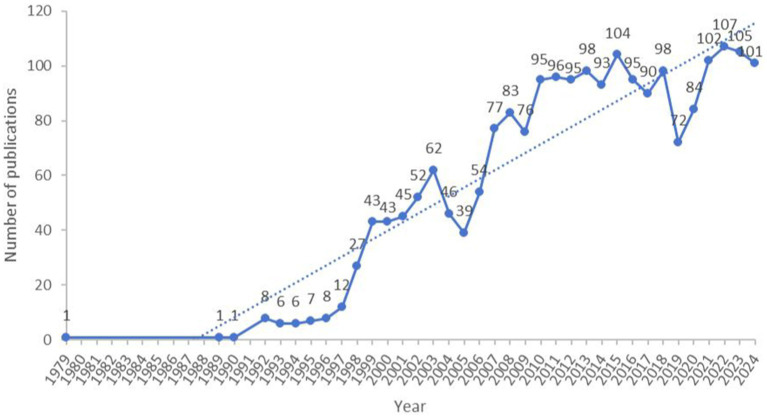
Annual publication volume of arterial compression hemostatic devices.

The research and publication volume on arterial compression hemostasis devices have shown an overall upward trend since 1979, particularly after the year 2000. From 1979 to 1999, the annual number of publications in this field exhibited a gradual increase, which can be regarded as the initial phase of research on arterial compression hemostasis devices. This period witnessed a gradual rise in interest and research activities related to this technology. The period from 2000 to 2010 was characterized by a rapid increase in publications, likely driven by technological advancements and the growing clinical demand. During this time, numerous new devices emerged, such as mechanical compression hemostasis devices [e.g., FemoStop (21) and CompressAR (22)], which provide continuous and adjustable pressure; bioabsorbable hemostasis devices [e.g., Angio-Sea (23) and Mynx (24)], which use biocompatible materials to seal puncture sites; inflatable tourniquets [e.g., TR Band (25)], which apply pressure through inflation to achieve hemostasis; and hemostatic dressings [e.g., QuikClot (26) and Celox (27)], which rapidly promote blood coagulation. These innovations significantly enhanced hemostatic efficacy and patient safety, thereby fueling the rapid market growth. From 2011 to 2020, the publication volume remained relatively stable. In the most recent years (2021–2024), the publication volume has been maintained at a relatively high level with a more stable growth rate. During this period, many new types of devices emerged and developed towards intelligence and precision. Future projections suggest that research in this field will continue to remain active, likely driven by ongoing technological advancements and increasing clinical demands. Overall, these devices have improved procedural efficiency and reduced complication rates in emergency settings. However, although there is growing academic interest in this trend, further studies are needed to evaluate the long-term clinical outcomes and cost-effectiveness of these devices across diverse patient populations. For a visual summary, see Figure 2.

### 3.2 Author analysis

**Figure 3:**
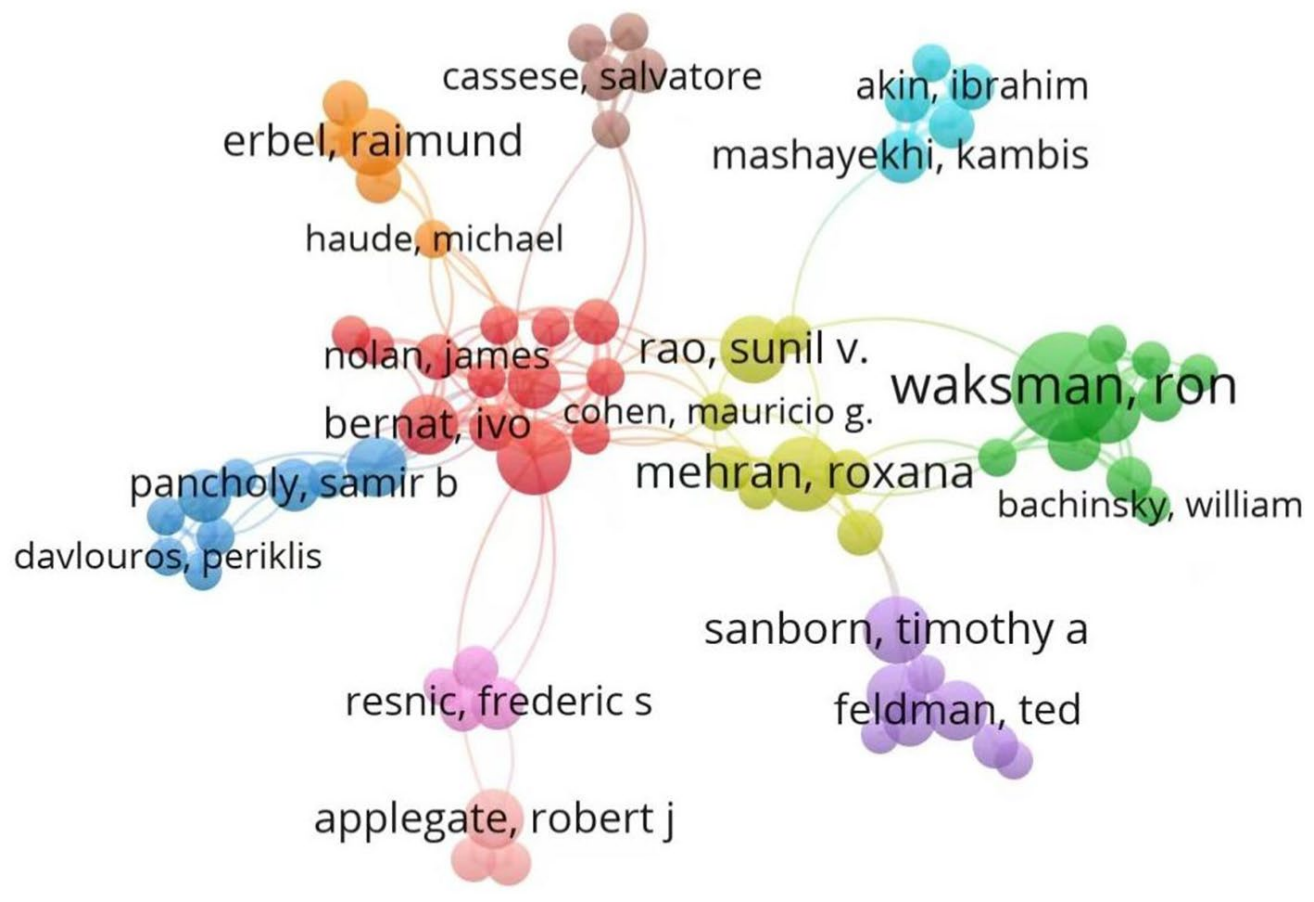
Network map of author collaboration in WOS and Scopus publications.

This study presents the top 10 authors with the highest number of publications in Table 1, based on the ranking of publication counts. Bernat, Ivo has the highest number of publications, with a total of 6 articles, followed by Silber, Sigmund with 5 articles per person. Sunil V Rao ranks first in both total citation counts and h-index. His research primarily focuses on the cardiovascular field, particularly on the prediction and stratification of ischemic and hemorrhagic risks following percutaneous coronary intervention (PCI). He has explored how risk assessment models and antiplatelet treatment strategies can improve the prognosis of patients after PCI (28). Additionally, he has participated in several studies related to arterial compression hemostasis devices, especially investigating the effectiveness of hemostasis devices after radial artery access for PCI. These studies provide important references for clinicians in selecting appropriate hemostasis devices and optimizing postoperative care strategies, which can help reduce the incidence of postoperative complications and enhance the quality of postoperative recovery for patients (29). Figure 3 presents a clustering map of author collaboration. It is noteworthy that the collaboration within the team is relatively close, with authors such as Nolan, James, Rao, Sunil V, and Cohen, Mauricio G having numerous connections with other nodes. However, collaboration between teams is relatively limited, indicating a need to strengthen inter-team cooperation. The lack of connections between teams may restrict overall efficiency and innovation, while sharing resources between different teams can enhance the level of research.

**Table 1:** Top 10 authors and institutions in arterial compression hemostatic device publications from 1979 to 2024.

Ranking	Foreign authors		Foreign institutions	
	Name	Publication volume	Title	Publication volume
1	Bernat, lvo	6	Department of Cardiology	108
2	Silber, Sigmund	5	Department of Radiology	85
3	Cannon, L	4	Division of Cardiology	54
4	Bachinsky, William	4	Department of Surgery	36
5	Zapien, M	4	Department of Vascular Surgery	23
6	Akin, lbrahim	4	Harvard University	20
7	Didonato, K	4	Department of Medicine	17
8	Applegate, Robertj	4	Deparment of Neurosurgery	17
9	Behnes, Michael	4	University of California System	14
10	Hinohara, T	4	Assistance Publique Hopitaux Paris (APHP)	13

### 3.3 Institutional analysis

**Figure 4:**
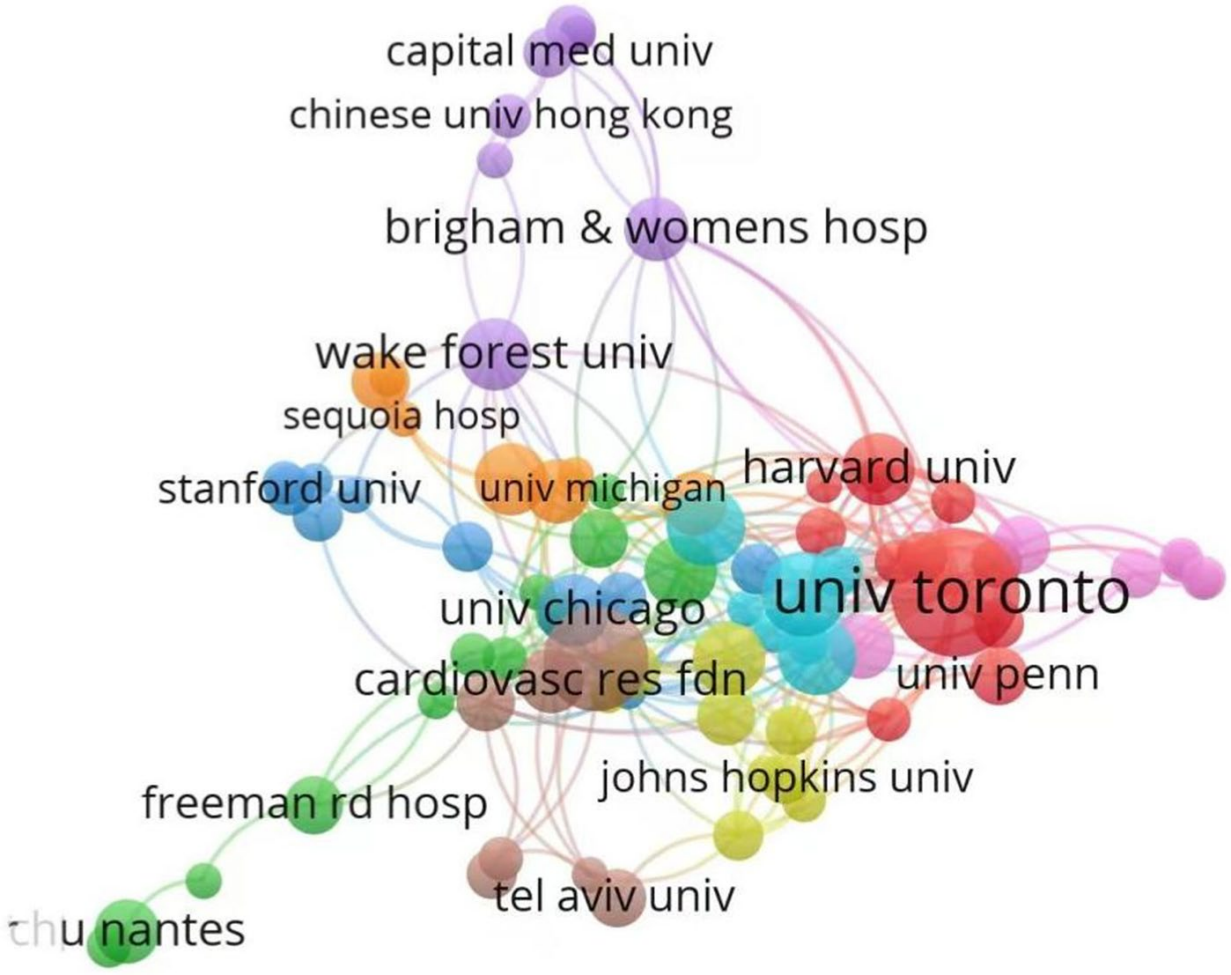
Inter-agency partnerships. The size of the circle shows the number of articles originating from the institution. The thickness of the connecting lines shows the strength of cooperation among institutions. Agencies that cooperate more frequently form color-coded clusters.

The academic standing of an institution within a given field can be evaluated through its annual scholarly output and the quality of its research achievements (19). As delineated in Table 1, the top 10 institutions ranked by publication volume are presented. The Department of Cardiology (108 publications), Department of Radiology (85 publications), and Division of Cardiology (54 publications) occupy the top three positions in terms of productivity. Notably, the leading institution, the Department of Cardiology, has accrued a total of 2,729 citations, underscoring its scholarly impact. Regarding centrality metrics—a critical indicator of influence, particularly in the context of arterial compression hemostat research—the most prominent institutions are the Department of Cardiology (0.17), Division of Cardiology (0.14), and Harvard University (0.09). Figure 4 visually represents the dynamic collaborative network among institutions. Nodes, color-coded to distinguish distinct collaborative clusters, vary in size to reflect an institution’s relative prominence or influence within the network. Interconnecting lines denote collaborative relationships, with thicker lines indicating stronger partnerships (19). The analysis reveals a pronounced geographical clustering pattern: institutions within the same cluster are predominantly concentrated in specific regions. European and American institutions exhibit a strong propensity for international collaboration, with Harvard University and the University of Toronto serving as pivotal hubs, maintaining extensive cross-border partnerships. Conversely, Chinese institutions prioritize domestic collaborations, demonstrating limited engagement with international counterparts. This geographical stratification vividly illustrates the regionalization inherent in academic collaboration, highlighting divergent strategic emphases across global research networks.

### 3.4 Visualization and analysis of keywords

#### 3.4.1 Representation of co-occurrence network of keywords

**Figure 5:**
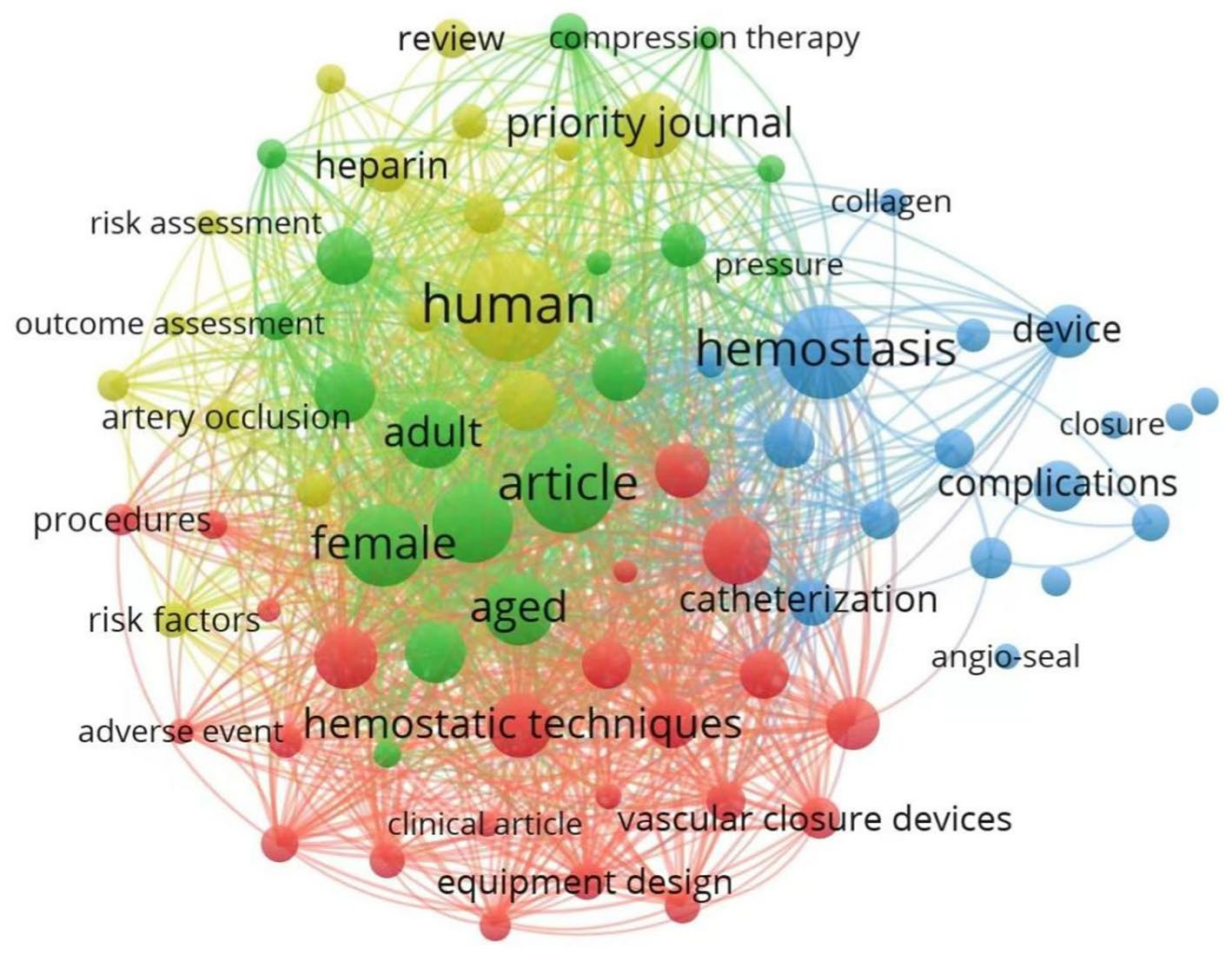
Co-occurrence map of key terms in arterial compression hemostatic device research based on VOSviewer.

The keyword nodes and their interconnecting lines within the co-occurrence network illustrate the degree of association between terms, with line thickness corresponding to the strength of correlations. Node size is proportional to keyword frequency, reflecting the academic prominence and influence of specific terms within the research domain (30). Figure 5 presents a knowledge graph constructed from keywords appearing in 91 or more publications within the international literature. Analysis of 7,724 identified keywords revealed core research dimensions through co-occurrence patterns. Table 2 enumerates the 25 keywords exhibiting the highest co-occurrence frequencies and centrality metrics. The five most frequent terms were “human” (918 instances), “article” (726), “hemostasis” (643), “female” (592), and “male” (575). These high-frequency keywords form an interconnected network that delineates critical aspects of arterial compression hemostasis research. This indicates that the research focus, gender differences, and academic dissemination of arterial compression hemostasis devices are hotspots in the research. Centrality analysis identified five pivotal terms: “cardiac catheterization” (0.09), “device” (0.07), “treatment outcome” (0.06), “bleeding” (0.06), and “female” (0.05), emphasizing the critical role of arterial compression devices in cardiac interventions and ongoing optimization efforts targeting therapeutic efficacy and risk mitigation.

**Table 2:** Top 25 most frequent keywords and highest centrality keywords.

Rank	Frequency	Keywords	Centrality	Keywords
1	918	human	0.09	cardiac catheterization
2	726	article	0.07	device
3	643	hemostasis	0.06	treatment outcome
4	592	female	0.06	bleeding
5	575	male	0.05	female
6	475	femoral artery	0.05	adult
7	473	aged	0.05	major clinical study
8	446	adult	0.05	heparin
9	410	treatment outcome	0.05	acetylsalicylic acid
10	400	hemostatic techniques	0.04	hemostasis
11	392	priority journal	0.04	male
12	385	major clinical study	0.04	priority journal
13	376	middle aged	0.04	middle aged
14	345	bleeding	0.04	false aneurysm
15	344	controlled study	0.04	heart catheterization
16	310	hematoma	0.04	angioplasty
17	303	vascular access	0.04	clinical trial
18	284	cardiac catheterization	0.03	human
19	284	percutaneous coronary intervention	0.03	aged
20	276	vascular closure device	0.03	controlled study
21	266	device	0.03	hematoma
22	263	catheterization	0.03	catheterization
23	253	punctures	0.03	punctures
24	246	false aneurysm	0.03	prospective study
25	242	heparin	0.03	randomized controlled trial

#### 3.4.2 Analysis of keyword clusters

**Figure 6:**
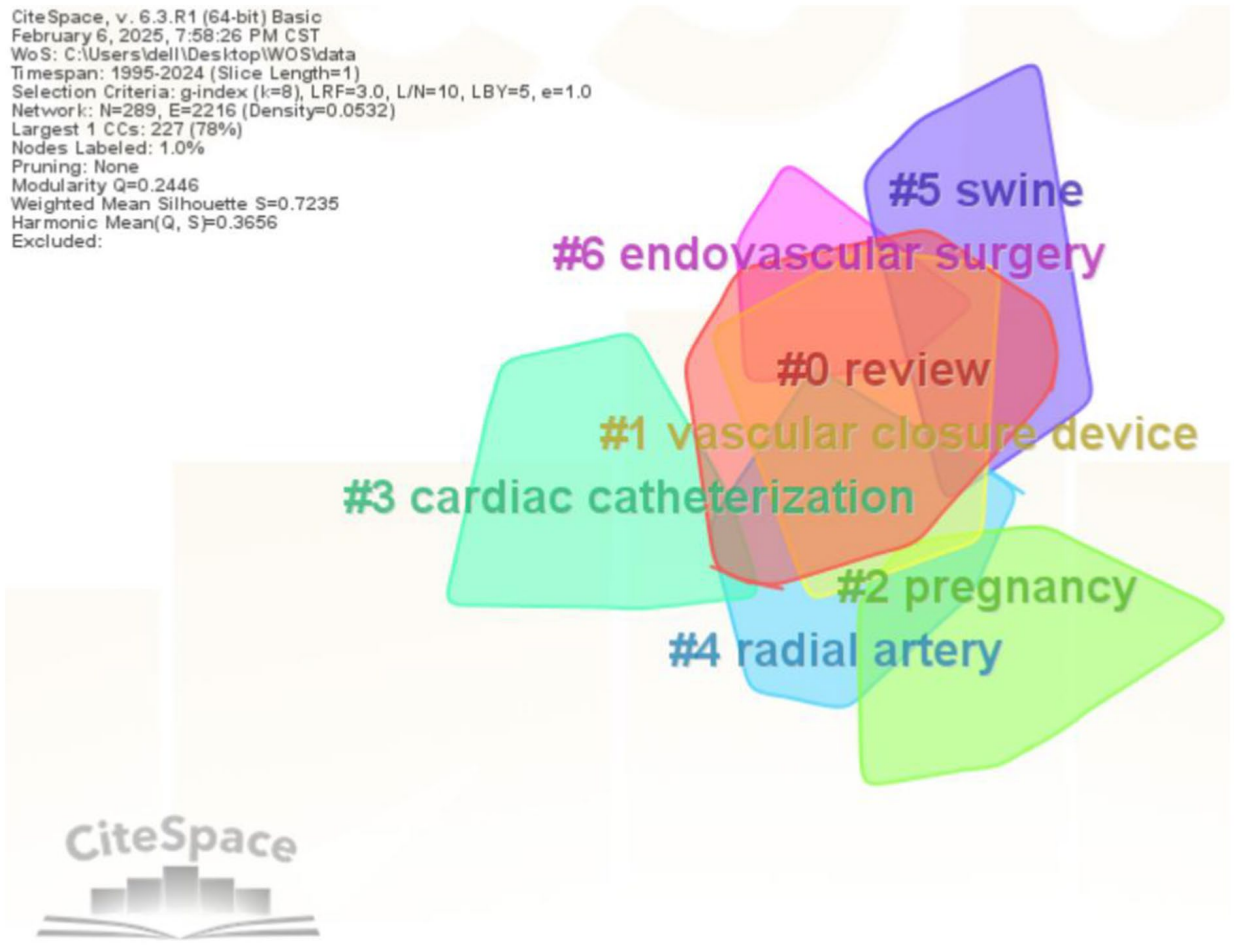
Keyword clustering network.

Cluster analysis is a data classification method based on similarity metrics for datasets lacking predefined categorical information, enabling the revelation of knowledge frameworks and their evolutionary patterns within specific domains (31). This study employed CiteSpace to conduct keyword cluster analysis and construct a keyword clustering map, systematically investigating core research hotspots and knowledge architecture within the field. The resulting map (Figure 6) comprises seven clusters. The modularity (Q-value) of 0.2446 indicates relatively weak cluster structure significance, while the average silhouette (S-value) of 0.7235 demonstrates high reliability and strong internal consistency within clusters (31). The seven identified clusters are: #0 review, #1 vascular closure device, #2 pregnancy, #3 cardiac catheterization, #4 radial artery, #5 swine, and #6 endovascular surgery. These clusters can be categorized into four thematic groups: (1) Cardiovascular interventional procedures (#3, #4, #6); (2) Hemostatic technologies and devices (#1); (3) Research models and reviews (#0, #5); (4) Special clinical application scenarios (#2). The largest cluster (#0 review) reveals a substantial increase in review literature on arterial compression hemostatic devices in recent years, reflecting intensified research efforts. Future developments in intelligent and personalized technologies are anticipated to enhance the clinical utility of these devices in vascular interventions, while simultaneously optimizing their safety profiles and cost-effectiveness. These findings indicate a deepening of research on arterial compression hemostatic devices in recent years.

#### 3.4.3 Keywords with citation bursts

**Figure 7:**
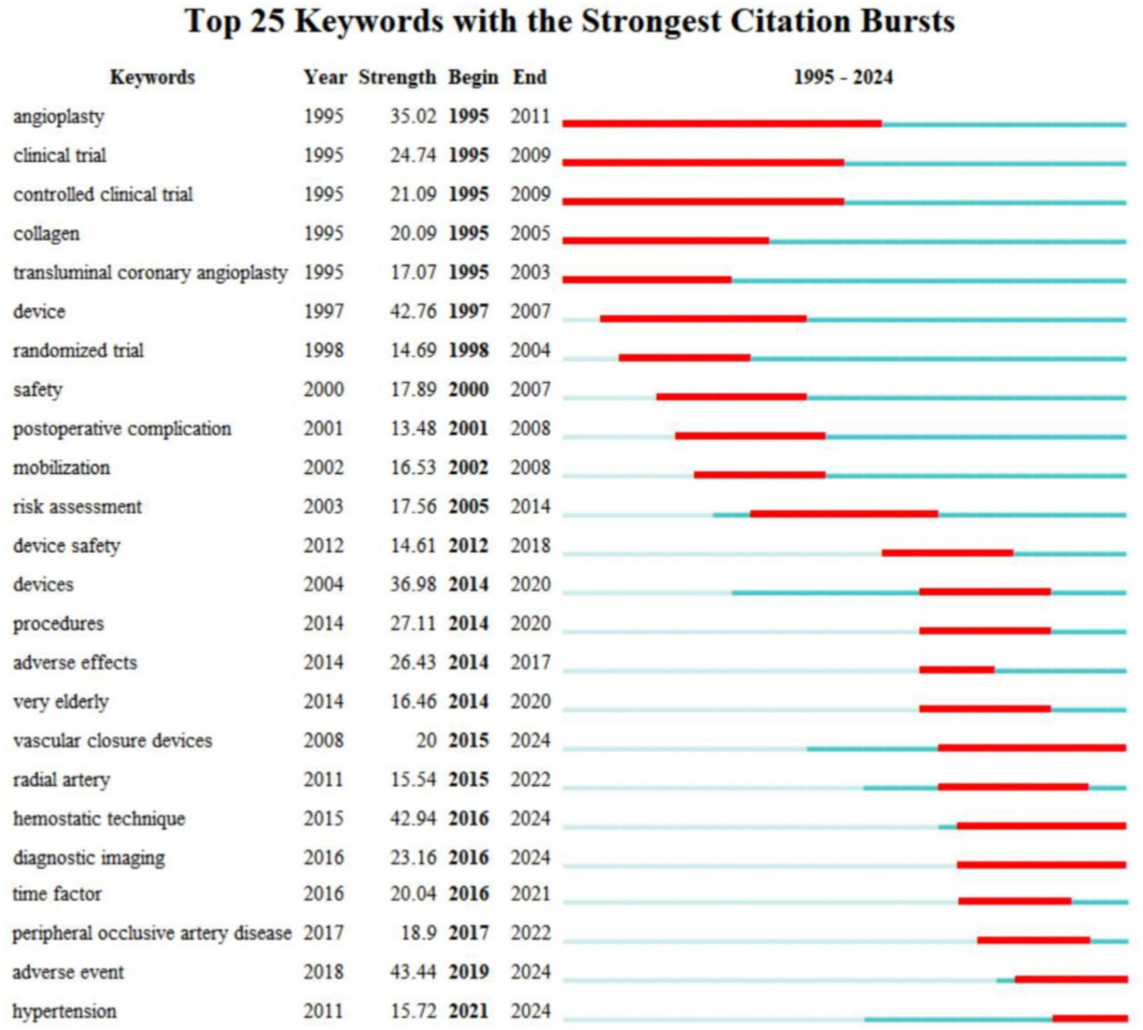
Top 25 keywords with the strongest citation bursts. Blue lines indicate time intervals. Red lines indicate periods of sudden increases in citation frequency.

Keyword bursts, defined as abrupt frequency increases of specific terms within defined timeframes, serve as indicators of emerging research frontiers (31). Figure 7 presents the top 25 keywords with citation bursts identified through CiteSpace (V6.2.R4) analysis of English publications. Blue segments denote temporal spans, while red segments highlight burst periods (31). The analysis reveals "angioplasty" as the most enduring keyword (1995-2011, burst strength: 35.02). High-intensity bursts include "adverse event" (43.44), "hemostatic technique" (42.94), and "device" (42.76). Early research emphasized long-term foci such as "angioplasty" 、"controlled clinical trial" and "collagen," while mid-phase studies concentrated on "risk assessment," "device safety," and "mobilization." Recent trends highlight "hypertension," "adverse event," "vascular closure devices," "diagnostic imaging," and "hemostatic technique," reflecting growing clinical emphasis on patient safety, therapeutic efficacy, and procedural comfort. This evolution manifests not only in technological advancements but also in optimized healthcare services and refined management protocols.

## 4 Discussion

### 4.1 Arterial compression hemostatic devices: a steady upward research trend

The prevalence of cardiovascular and cerebrovascular diseases is rising worldwide, leading to increased morbidity and mortality rates (32, 33). The rise of interventional therapies has spurred research into arterial compression and postoperative complication management (34). However, hemostasis techniques are still developing, and research on hemostasis devices and technological improvements is limited (35). It is crucial to outline the research landscape for hemostasis techniques and devices in arterial interventional therapy.

Arterial compression hemostatic devices, also known as vascular closure devices (VCDs), are used in interventional radiology to achieve hemostasis after procedures involving arterial access (10). This study conducted a bibliometric analysis of English-language literature from the Web of Science and Scopus databases to provide a comprehensive overview of the current status, emerging trends, academic collaborations, and future directions in research on arterial hemostatic devices (36). The findings reveal that publications on this topic have significantly increased since 1979, particularly in the post-2000 era, driven by close collaborations among research institutions. Over time, various hemostatic devices have been developed. From 1979 to 1999, the number of publications in this field gradually increased, marking the initial stage of research on arterial compression hemostasis. During this period, manual compression combined with sandbag pressure was the primary method, supplemented by simple fixation devices such as bandages or splints. This stage was characterized by the absence of specialized hemostatic devices, reliance on manual pressure application by healthcare providers, and prolonged operation times with poor stability. The period from 2000 to 2010 was marked by rapid growth in publications, likely due to the emergence of mechanically adjustable hemostatic devices. Representative devices included mechanically regulated hemostatic devices (e.g., radial artery hemostatic devices with a screw handle for pressure adjustment (37), incorporating elastic components and pads for stable compression) and femoral artery hemostatic devices (38)(e.g., structures combining a scaffold base with compression support columns). The technological hallmark of this era was simple mechanical structures and low costs, although these devices relied on manual pressure adjustment, which posed risks of displacement and potential hemostasis failure. From 2011 to 2020, the number of publications remained relatively stable, reflecting a phase of multifunctional improvements. Prominent devices during this period included produced YM-GU series devices (e.g., the YM-GU-1229-type arterial hemostatic device, which demonstrated short hemostasis times and fewer complications, becoming a commonly utilized device in post-interventional procedures) and balloon-type and non-balloon-type devices (with balloon-type devices utilizing inflation systems for pressure adjustment (39) and non-balloon-type devices employing screw handles or compression plate structures to meet diverse clinical needs (39)). Innovation during this period focused on enhancing comfort (e.g., detachable compression pads) and optimizing fixation devices to reduce displacement risks. In recent years (2021–2024), the number of publications has remained at a high level with stable growth, marking the entry into an era of intelligent and precise hemostatic devices. This period has seen the emergence of numerous novel devices, research on hemostasis devices made of biocompatible materials has gradually increased, such as the Multi-crosslinking nanoclay/oxidized cellulose hydrogel bandage (40), which possesses strong mechanical strength, antibacterial properties, and adhesive capabilities for emergency hemostasis. The Manta Vascular Closure Device (41), a novel collagen-based vascular closure device, has also demonstrated excellent hemostatic effects; The evolution of hemostatic devices has been marked by the increasing adoption of advanced technologies, including dual-fixation hemostatic devices, automated compression systems, and personalized or high-end products featuring intelligent functionalities (e.g., pressure sensing (42)) and customization tailored to diverse vascular anatomies. Certain devices further integrate temperature monitoring capabilities (43). These innovations have demonstrated efficacy in reducing immobilization durations, mitigating complication rates, and alleviating the operational burden on healthcare professionals. Collectively, the technological progression across four distinct phases reflects a paradigm shift from manual intervention to mechanical regulation and, ultimately, to intelligent management systems. Recent advancements have prioritized three key domains: stability enhancement (e.g., dual-fixation mechanisms), automation via sensor-driven feedback systems, and personalized adaptability (e.g., multi-angle adjustability). Such refinements have substantially optimized hemostatic outcomes while concurrently improving patient comfort and clinical efficiency.

Analyzing the most prolific authors can help identify the core themes within the research field of arterial compression hemostatic devices (19). Among the internationally most academically influential authors, Bernat, Ivo from the Czech Republic has published the highest number of papers. His primary research focus lies in cardiovascular diseases, particularly transradial coronary interventions (TRI) and related technologies. His studies have compared the efficacy of various compression hemostatic devices in achieving hemostasis following transradial coronary interventions. For instance, one of his studies compared the TR Band with manual compression methods, revealing that the TR Band offers certain advantages in terms of hemostasis time and safety (44). He has also explored novel hemostatic techniques, such as the method of achieving radial artery recanalization through brief compression of the contralateral ulnar artery, which has been proven effective and safe in patients undergoing low-dose and very low-dose heparin therapy (45). Additionally, he has contributed to the advancement of distal radial artery puncture techniques, participating in the development and design of distal radial artery puncture hemostatic devices. These innovative devices enable precise compression at the puncture site, reducing the risk of radial artery occlusion (RAO) (46). Such research not only optimizes hemostatic outcomes and minimizes complications but also enhances patient comfort and nursing convenience, expands the applicable patient population, and provides more treatment options, thereby driving the progress of interventional therapy technologies. Institutions have made significant contributions to this field on an international scale. Notably, the Department of Cardiology, a leading institution, has been cited a total of 2,729 times, underscoring its academic influence. European and American institutions have established a robust network of partnerships, including international and inter-institutional collaborations, with Harvard University and the University of Toronto serving as key hubs and maintaining extensive cross-border relationships. In contrast, Chinese institutions prioritize domestic collaborations, with limited engagement with international peers. It is recommended to establish a shared database to support research on the application of arterial hemostatic devices and provide academic support for future studies.

### 4.2 Research hotspots and future trends

Through the analysis of keyword frequency and centrality in the study of arterial compression hemostatic devices, it is possible to identify the research hotspots in this field (19). The findings reveal that the research primarily focuses on ‘human’ (918), ‘article’ (726), ‘hemostasis’ (643), ‘female’ (592), and ‘male’ (575). This indicates that the key areas of interest in the study of arterial compression hemostatic devices include their application focus, gender differences, and academic dissemination. The keyword ‘human,’ which appears most frequently, underscores that the research is predominantly centered on human applications. The primary objective of arterial compression hemostatic devices is to assist in controlling bleeding in medical settings, particularly during surgeries, trauma, or emergency situations. The research emphasis lies in optimizing the efficacy and safety of these devices in human use. The keyword ‘article’ highlights that the research is primarily disseminated through academic publications or literature, suggesting that a systematic body of knowledge has been established in this field. This academic exchange continues to drive technological advancements. The keyword ‘hemostasis,’ which represents the core function of arterial compression hemostatic devices, indicates that the research is focused on achieving rapid and effective hemostasis. This keyword reflects the central goal of the research, which is to enhance hemostatic efficiency through technological innovation, thereby reducing blood loss and associated complications. Notably, its connections to gender-specific terms (“female” and “male”) highlight emerging research directions investigating anatomical variations’ impact on hemostatic efficacy. Key findings include prolonged compression durations in females due to smaller radial artery diameters (47) and pressure transmission discrepancies in males associated with higher muscle density (48). This gender-differentiated approach is driving a paradigm shift from universal to personalized device design. However, methodological limitations persist, as only 34.7% of gender-related studies implemented paired experimental designs, potentially compromising result generalizability. Future research will focus on the multifaceted optimization of hemostatic technologies. On one hand, by strengthening multicenter clinical trials and integrating biomechanical modeling and real-time monitoring technologies, researchers aim to deeply analyze the dynamic relationship between human physiological characteristics and hemostatic efficacy, thereby providing theoretical support for the development of intelligent hemostatic devices. On the other hand, efforts will be directed toward developing more personalized hemostatic equipment tailored to different genders, ages, and physiological characteristics, addressing diverse clinical needs. Concurrently, further investigation into hemostatic mechanisms will optimize the technical performance of hemostatic devices, enhancing their efficiency and safety. Additionally, emphasis will be placed on advancing clinical application research of hemostatic devices in emergency medical and surgical settings, comprehensively evaluating their effectiveness, and driving the continuous progress of hemostatic technologies. Centrality analysis has identified five key terms: “cardiac catheterization” (0.09), “device” (0.07), “treatment outcome” (0.06), “bleeding” (0.06), and “female” (0.05). This study centers on “cardiac catheterization” as a critical medical scenario, focusing on the design of hemostatic devices and their efficacy in controlling bleeding. Specifically, the research targets female subjects to explore the impact of gender factors on hemostatic outcomes. Furthermore, the study highlights technological innovations in hemostatic devices and conducts a comprehensive evaluation of their clinical efficacy, reflecting a complete research chain from theory to practice. Advancement trajectories are anticipated to focus on personalized device architectures, intelligent technology integration, and expanded clinical validation to enhance both functional performance and patient outcomes. This analytical framework demonstrates how co-occurrence network topology maps research priorities and knowledge gaps, providing empirical guidance for strategic research planning in medical device innovation.

Keyword clustering is a method that categorizes and integrates closely related keywords based on their co-occurrence frequency. Clusters with a larger number of keywords are typically assigned smaller numerical labels (19). The five largest clusters identified are #0 review, #1 vascular closure device, #2 pregnancy, #3 cardiac catheterization, and #4 radial artery. The arterial compression hemostatic device, as the core subject of the vascular closure device cluster (#1), exhibits multidimensional associations in terms of its technical principles and clinical applications across these clusters. This device plays a pivotal role in post-procedural hemostasis following cardiac catheterization (#3), particularly in association with radial artery access techniques (#4). The transradial approach, known for its minimal invasiveness and rapid recovery, has led to the accumulation of substantial evidence supporting the use of arterial compression hemostatic devices in radial artery hemostasis. In the context of pregnancy-related studies (#2), the device’s unique value lies in its adaptability to address coagulation abnormalities and anticoagulation needs in pregnant women. Notably, systematic reviews (#0) have highlighted the comparative advantages of this device across various clinical scenarios: it outperforms traditional manual compression in reducing complications and shortening immobilization time, though its use may be limited in patients with extreme obesity or peripheral vascular disease. This cross-cluster connectivity demonstrates that research on arterial compression hemostatic devices has expanded beyond single-device innovation to encompass perioperative management optimization and adaptability for special populations, forming a multidimensional research network centered on vascular closure with multidisciplinary applications.

Keyword burst refers to the phenomenon where the citation frequency of a specific keyword undergoes significant changes within a particular time period, reflecting its rising or declining trend (19). Based on burst analysis of keywords, the top 25 keywords with the highest burst intensity were identified, among which "Angioplasty" exhibited the longest duration and a sharp increase in citations in 1995. This phenomenon likely reflects the growing attention to angioplasty during that period. Angioplasty is a minimally invasive procedure used to treat vascular stenosis or occlusion, commonly applied to coronary arteries (the primary blood vessels supplying the heart) or other peripheral vessels (49). Arterial compression hemostatic devices serve as essential tools in post-angioplasty care. Since its first clinical application in 1977, angioplasty has continuously evolved, with the advent of drug-eluting stents significantly reducing restenosis rates (50). Continuous technological innovation, broad clinical demand, and interdisciplinary research are likely the primary reasons for the sustained focus on angioplasty. The surge in citations in 1995 can be attributed to several factors: the widespread adoption of stent technology (e.g., in 1994, the first bare-metal stent, the Palmaz-Schatz stent (51), received FDA approval, marking the transition of angioplasty into the stent era; in 1995, clinical research and application of stent technology rapidly increased, driving the publication and citation of related literature), the publication of large-scale clinical trials (e.g., in the mid-1990s, results from multiple large-scale clinical trials comparing stents with balloon angioplasty, such as the STRESS (52) and BENESTENT trials (53), were published, demonstrating the superiority of stents in reducing restenosis rates and improving clinical outcomes, which garnered significant academic attention), shifts in research focus (e.g., in the early 1990s, angioplasty research primarily centered on balloon technology (54), but by 1995, the focus gradually shifted to stent technology (55), spurring the generation and citation of numerous new studies), and the influence of academic conferences and guidelines (e.g., in the mid-1990s, major cardiovascular conferences such as the American College of Cardiology Annual Meeting (ACC) (56) and the European Society of Cardiology Annual Meeting (ESC) (57) frequently discussed the latest advancements in angioplasty, and updates to related guidelines also contributed to increased citation of literature). This period marked the transition of angioplasty from the balloon era to the stent era, representing a significant milestone in the treatment of cardiovascular diseases.

The terms “controlled clinical trial” and “collagen” emerged as early focal points in 1995, with burst intensities of 21.09 and 20.09, respectively. These bursts reflect two critical trends in the development of arterial compression hemostatic devices: the standardization of research methodologies (the widespread adoption of controlled clinical trials) and advancements in material science (the application of collagen in hemostatic materials). The emergence of these two burst terms marks a significant milestone in the transition of arterial compression hemostatic devices from theoretical research to clinical application. The analysis suggests that the reasons for these bursts may be attributed to the growing emphasis on Evidence-Based Medicine (EBM) (58) in the medical research field around 1995. Controlled clinical trials, regarded as the “gold standard” for evaluating the efficacy of medical devices, drugs, and treatment methods, were widely promoted and applied during this period. As a novel medical device, the safety and effectiveness of arterial compression hemostatic devices required validation through rigorous controlled clinical trials, which likely contributed to the emergence of “controlled clinical trial” as a burst term in 1995. Collagen, a natural biomaterial with excellent biocompatibility and hemostatic properties (59), saw significant advancements in its application within the medical device field around the same time, particularly in hemostatic materials and wound healing. This technological breakthrough or research focus likely propelled “collagen” to become a burst term. Together, these developments underscore the intersection of methodological rigor and material innovation in the evolution of arterial compression hemostatic technology.

The terms “risk assessment,” “device safety,” and “mobilization” emerged as mid-term hotspots with prolonged durations, reaching their burst intensities in 2005, 2012, and 2002, respectively. The analysis suggests that the burst of “risk assessment” in 2005 marked the integration of risk management as a core component in the development and production of medical devices. Regulatory agencies worldwide, such as the FDA and CE, intensified their requirements for risk assessment of medical devices during this period. As arterial compression hemostatic devices directly interact with the human body, their potential risks (e.g., infection, thrombosis, tissue damage) necessitated systematic evaluation. The widespread adoption of these devices revealed associated risks and adverse events, prompting researchers and manufacturers to prioritize risk assessment. Consequently, risk management became a central focus in medical device development, driving “risk assessment” to prominence as a hotspot. The burst of “device safety” in 2012 reflected heightened attention to the safety of medical devices, particularly in the prevention of adverse events and technological improvements. Around this time, the global medical device industry reached a peak in its focus on safety. As arterial compression hemostatic devices became more widely used, related adverse events (e.g., tissue necrosis due to excessive compression or bleeding due to insufficient compression) were increasingly reported, prompting researchers and regulators to emphasize device safety. The introduction of new materials and design technologies, such as intelligent pressure control systems (60), further highlighted safety as a critical area of research and innovation. Additionally, regulatory agencies worldwide strengthened their scrutiny and oversight of device safety, solidifying “device safety” as a key hotspot. The burst of “mobilization” in 2002 signified a shift in the design philosophy of medical devices, emphasizing patient comfort and mobility. During this period, the design paradigm of medical devices transitioned from “immobilization” to “mobilization,” (61) prioritizing patient comfort and freedom of movement. Traditional arterial compression hemostatic devices often restricted patient mobility, whereas mobile designs (e.g., lightweight, adjustable devices) enhanced patient comfort and postoperative recovery. Advances in materials and miniaturization technologies facilitated the development of lighter and more flexible devices, driving the trend toward mobilization. Furthermore, the expanding application of arterial compression hemostatic devices in diverse settings (e.g., emergency care, battlefield medicine) underscored the importance of mobile designs in meeting varied clinical needs. This evolution in design philosophy propelled “mobilization” to the forefront as a significant research focus.

Furthermore, in recent years, the research landscape has continued to expand, with terms such as “hypertension,” “vascular closure devices,” “diagnostic imaging,” “hemostatic technique,” and “adverse event” (with the highest burst intensity) showing sustained momentum in 2024, indicating their potential as future research directions. Studies in the field of arterial compression hemostatic devices are currently centered around five key hotspots: addressing the specific needs of hypertensive patient populations, technological innovations in vascular closure devices, the integration of diagnostic imaging with precision hemostasis, the diversified development of hemostatic techniques, and the risk management of adverse events. The global aging population and the increasing prevalence of cardiovascular diseases have led to a surge in surgical procedures, driving the demand for efficient and safe hemostatic solutions. Hypertensive patients, with their heightened vascular fragility, face greater challenges in postoperative hemostasis. Research efforts are focused on optimizing the pressure control, material adaptability, and complication prevention of hemostatic devices, while balancing hemostatic efficacy with vascular protection, thereby advancing the development of personalized hemostatic solutions. The widespread adoption of minimally invasive surgeries has positioned vascular closure devices as alternatives to traditional compression hemostasis. Innovations in biocompatible materials and intelligent designs have enhanced their safety and convenience. The incorporation of diagnostic imaging technologies has enabled real-time monitoring of the hemostatic process and postoperative outcome evaluation, reducing procedural errors and supporting the early detection of complications. The development of novel hemostatic materials (e.g., hemostatic gels, nanomaterials) (62) and intelligent devices has further reduced reliance on mechanical compression, improving hemostatic efficiency and safety. Additionally, clinical data analysis has been employed to understand the mechanisms underlying adverse events, optimizing compression duration, pressure thresholds, and material biocompatibility to effectively mitigate complications such as local tissue damage, nerve compression, and infection. Industry competition and technological barriers have driven companies to enhance their competitiveness through technological innovation and patent strategies, while meeting the stringent safety requirements of regulatory agencies, thereby fostering refined research approaches. The sustained momentum of these hotspots reflects the demand for comprehensive innovation across the entire chain of arterial compression hemostatic device development, from foundational technologies to clinical applications. Future research will increasingly integrate interdisciplinary technologies (e.g., materials science, artificial intelligence) to address the challenges of complex clinical scenarios.

Visual analysis based on keywords reveals progress in hemostasis technique research across various fields, driven by the widespread adoption of interventional strategies. However, social network mapping is lacking (63), as it does not provide sufficient precision to predict future research foci and pathways for arterial hemostasis techniques. It is recommended to enhance communication and collaboration, explore arterial hemostasis techniques further, improve accessibility (64), advance hemostasis devices, review social network maps comprehensively, and establish standardized guidelines for the use of arterial hemostasis agents.

## 5 Limitations

To encapsulate, the current investigation harnessed the analytical capabilities of VOSviewer and CiteSpace software to dissect the prevailing landscape, nascent thematic developments, and prospective trajectories within the domain of arterial tourniquet devices. It is imperative to underscore two principal constraints inherent to our methodology. Initially, the plethora of synonyms and associated terminologies pertaining to arterial compression hemostasis devices were not subjected to a thorough synthesis and scrutiny. Subsequently, the exclusive reliance on the Web of Science (WOS) and Scopus databases for literature synthesis potentially imparts a confounding bias to our findings. In pursuit of augmenting the profundity and expanse of forthcoming research endeavors on arterial compression hemostasis devices, we advocate for the deployment of sophisticated visualization instruments and the integration of data from a spectrum of databases to achieve a more panoramic dissection of research trends. Additionally, the cultivation of collaborative efforts across international research consortia, coupled with the execution of randomized controlled trials assessing diverse hemostasis techniques for arterial compression tourniquet devices, is deemed indispensable for enhancing the practical utility and efficacy of tourniquets. These measures are pivotal for bolstering the integrity of research outcomes and catalyzing innovation within the arena of arterial hemostasis devices.

## 6 Conclusion

This study employed bibliometric analysis tools, namely VOSviewer 1.6.18 and CiteSpace 6.2.r4 software, to analyze relevant literature, thereby unveiling a promising and rapidly evolving research field. This field is dedicated to exploring the application of arterial compression hemostasis devices in cardiovascular and cerebrovascular diseases, with the aim of identifying emerging research directions and providing new perspectives for subsequent studies. Since 2000, research on arterial compression hemostasis devices has been continuously improving in both quality and quantity. Currently, the core issues in this field focus on optimizing hemostasis techniques and developing new types of devices. The emerging trends are concentrated on the integration of intelligent and precise technologies, personalized design and biomaterial innovation, interdisciplinary collaboration and expansion of clinical scenarios, as well as risk prevention and management of adverse events. Future research should address the limitations of existing data, strengthen clinical trials, and continue to explore new materials (such as biocompatible materials) and new technologies (such as smart sensors) to further enhance the performance of hemostasis devices. The above conclusions are based on a systematic analysis of institutions, authors, and keywords, which has comprehensively deepened the understanding of the field and provided important guidance for future research to cope with the increasing prevalence of cardiovascular and cerebrovascular diseases.

## Data Availability

The original contributions presented in the study are included in the article/supplementary material, further inquiries can be directed to the corresponding author.
